# Fhit-deficient normal and cancer cells are mitomycin C and UVC resistant

**DOI:** 10.1038/sj.bjc.6602058

**Published:** 2004-10-19

**Authors:** M Ottey, S-Y Han, T Druck, B L Barnoski, K A McCorkell, C M Croce, C Raventos-Suarez, C R Fairchild, Y Wang, K Huebner

**Affiliations:** 1 Department of Microbiology-Immunology, Kimmel Cancer Center, Jefferson Medical College, Philadelphia, USA; 2 Department of Medicine, Kimmel Cancer Center, Jefferson Medical College, Philadelphia, USA; 3 Oncology Drug Discovery, Bristol-Myers Squibb Pharmaceutical Research Institute, Princeton, NJ, USA; 4 Department of Radiation Oncology, Kimmel Cancer Center, Jefferson Medical College, Philadelphia, USA

**Keywords:** Fhit protein, UVC resistance, mitomycin C resistance, DNA damage checkpoint, Fhit deficiency

## Abstract

To identify functions of the fragile tumour suppressor gene, *FHIT*, matched pairs of Fhit-negative and -positive human cancer cell clones, and normal cell lines established from Fhit −/− and +/+ mice, were stressed and examined for differences in cell cycle kinetics and survival. A larger fraction of Fhit-negative human cancer cells and murine kidney cells survived treatment with mitomycin C or UVC light compared to matched Fhit-positive cells; ∼10-fold more colonies of Fhit-deficient cells survived high UVC doses in clonigenic assays. The human cancer cells were synchronised in G1, released into S and treated with UVC or mitomycin C. At 18 h post mitomycin C treatment ∼6-fold more Fhit-positive than -negative cells had died, and 18 h post UVC treatment 3.5-fold more Fhit-positive cells were dead. Similar results were obtained for the murine −/− cells. After low UVC doses, the rate of DNA synthesis in −/− cells decreased more rapidly and steeply than in +/+ cells, although the Atr–Chk1 pathway appeared intact in both cell types. UVC surviving Fhit −/− cells appear transformed and exhibit >5-fold increased mutation frequency. This increased mutation burden could explain the susceptibility of Fhit-deficient cells *in vivo* to malignant transformation.

*FHIT* gene structure and protein expression have been examined in detail in many types of cancers (for a review, see Huebner and Croce, 2003). Fhit expression is reduced or absent in the majority of human cancers due to genetic or epigenetic modification, though point mutations are very rare. The Fhit signalling pathway is of interest because the *FHIT* gene, in mouse and human, spans an active common chromosomal fragile site, that is susceptible to DNA damage due to intrinsic or extrinsic agents, including inhibitors of DNA replication, such as aphidicolin, the agent used to induce expression of common fragile sites. Replacement of Fhit in most Fhit-deficient cancer cells suppresses tumorigenicity (Siprashvili *et al*, 1997; Ishii *et al*, 2001; Sevignani *et al*, 2003) and *FHIT*-viral gene therapy, using adeno or AAV viral vectors, prevents and reverses carcinogen-induced gastric cancers in Fhit-deficient mice (Dumon *et al*, 2001; Ishii *et al*, 2003). Recombinant mice carrying one or two inactivated Fhit alleles are viable and long-lived but show increased rates of spontaneous and carcinogen-induced cancers (Fong *et al*, 2000; Zanesi *et al*, 2001); thus, Fhit-deficient mice are excellent models for preclinical studies of tumour development, prevention and therapy. From the extensive studies of the human and murine *FHIT* loci since its 1996 discovery, it is known that Fhit overexpression can cause death of Fhit-deficient cells through apoptosis (Ji *et al*, 1999; Sard *et al*, 1999; Dumon *et al*, 2001), while normal and Fhit-sufficient cells are unharmed; some details of the intrinsic apoptotic pathway activated in Fhit-positive cells have been reported (Ji *et al*, 1999; Dumon *et al*, 2001; Ishii *et al*, 2001; Sevignani *et al*, 2003).

Nevertheless, in the absence of confirmed interacting proteins, it has been difficult to define downstream effectors of Fhit signalling. We have used both human and mouse, Fhit-positive and -negative cells *in vitro*, to compare phenotypes of the two genotypes after exposure to various stressful conditions, such as low serum, detachment from substrate, hypoxia, irradiation and genotoxic drugs. We have observed that exposure to mitomycin C and UVC induces a phenotypic difference between Fhit-deficient and -sufficient cells, with the deficient cells showing a striking survival advantage after both treatments. To define the phenotypic difference in detail, with the elucidation of the Fhit signal pathway as the ultimate goal, we have performed survival, clonigenicity, cell cycle kinetics, rate of DNA synthesis and mutation rate analyses after exposure to mitomycin C and UVC treatments.

## MATERIALS AND METHODS

### Establishment and maintenance of cells

M1 Fhit −/− and +/+ kidney cells were established in culture from whole mouse kidney. Mice were killed using CO_2_ and dissected to obtain the kidney in accordance with guidelines of the TJU IACUC. Kidney tissue was disaggregated in RPMI medium (GIBCO, Carlsbad, CA, USA) supplemented with 10% FBS and 100 *μ*g ml^−1^ gentamicin. The cells were subcultured when they reached confluence. NIH Swiss kidney (Fhit +/+) cells were maintained in MEM (GIBCO) supplemented with 10% FBS and 100 *μ*g ml^−1^ gentamicin. The mouse cell lines showed near diploid karyotypes at passage 25 or earlier; the −/− cell line had an average of 42.6 chromosomes (range 41–44, with two marker chromosome) the +/+ cell line showed two populations with 60% of cells with an average of 43 chromosomes with a few markers and 40% of cells hypotriploid (average chromosome number 72, with 0–7 markers). MKN74 cells are a human gastric cancer cell line lacking Fhit expression. The clones used in these experiments are A66 and A116 (Fhit +) and E4 (empty vector) (Siprashvili *et al*, 1997). Fhit −/− UV1 cells were established from a surviving clone after treatment with 30 J m^−2^ UVC. These cells appear transformed and grow to a saturation density five-fold greater than the saturation density of the Fhit −/− parental cells. H1299, a lung tumour cell line stably transfected with the ecdysone-inducible vector pVgRXR (Invitrogen, Grand Island, NY, USA) was obtained from Dr Jennifer Pietenpol. These cells were then stably transfected with pIND-Fhit and clones tested for expression of Fhit before and after induction with 1 *μ*M Ponasterone A. Western blotting of clone D1 showed no Fhit expression without Ponasterone A and a steady increase after induction.

### Determination of cell growth (MTS assay)

*In vitro* cytotoxicity was assessed by 3-(4,5-dimethylthiazol-2-yl)-5-(3-carboxymethoxyphenyl)-2-(4-sulphenyl)-2H-tetrazolium, inner salt (MTS) assay, as previously described (Lee *et al*, 2001). Cells were seeded in microtitre plates and 24 h later drugs were added and serially diluted. The cells were incubated at 37°C for 72 h, at which time MTS and phenazine methosulphate were added. The absorbance was read at 492 nm, and the resulting OD was proportional to the number of viable cells.

### Induction of apoptosis (TUNEL analysis)

Fhit-positive and -negative cells were seeded in six-well plates, and the next day treated with drugs for 24 h. Cells were collected by trypsinisation and evaluated for apoptosis by TUNEL assay (APO-Direct Kit, BD Biosciences Pharmingen, San Diego, CA, USA). Data acquisition and analysis was performed on a FACScalibur flow cytometer from Beckton and Dickinson. Doublet discrimination gates were set prior to analysis using the Cellquest software, also from Beckton and Dickinson.

### Stress induction

*Mitomycin C*: Cells were seeded in 100 mm dishes, and allowed to attach and cycle for 16–24 h. Cells were then treated with medium supplemented with 2 or 5 *μ*M mitomycin C for specified times. *UVC irradiation*: Using a 254 nm UVC lamp (ULTRA LÛM, Inc, Paramount, CA, USA) which was calibrated each time before using, cells were exposed to 60 J m^−2^ UVC for some experiments; lower doses, 3–30 J m^−2^, were used for specified experiments.

### Survival and cell cycle kinetics assays

For survival assays, cells were counted, seeded in 100 mm dishes and left to grow for 16–24 h. Cells were either left untreated or exposed to UVC or mitomycin C and incubated for the specified periods. Attached cells were trypsinised, counted and compared at the collection time points. In some experiments, viable cells (attached and floating) were detected by Trypan blue dye exclusion after UVC exposure. Results of these experiments represent the percent of treated cells excluding Trypan blue in comparison to untreated cells. For assessment of clonigenicity, cells were seeded to 100 mm dishes and left to grow for 16–24 h. Cells were untreated or exposed to UVC or mitomycin C and then trypsinised, counted and re-seeded to 100 mm dishes at specified cell densities and incubated for up to 18 days for colony formation.

### Flow cytometry analysis

Cells were treated with UVC, mitomycin C or left untreated. At specified times, cells were collected by trypsinisation, washed with PBS and re-suspended in cold 70% ethanol. Fixed cells were stored at −20°C overnight. For analysis, cells were spun down, washed in PBS and suspended in propidium iodide (PI)/Triton X-100 staining solution (0.1% Triton X-100, 20 *μ*g ml^−1^ PI, 0.2 mg ml^−1^ of DNase-free RNase A (in PBS)). Cells were stained for 30 min at room temperature and flow cytometric analysis was performed. For S-phase BrdU labelling, cells were pulsed for 1 h with 10 *μ*M BrdU (Sigma), then treated or left untreated and incubated for the specified times. Cells were collected and fixed in cold 70% ethanol. Fixed cells were treated in denaturing buffer (2 M HCl), washed (PBS, 0.5% BSA) and incubated for 20 min at room temperature in anti-BrdU monoclonal antibody (Pharmingen, San Diego, CA, USA). Cells were washed, resuspended in PI (10 *μ*g ml^−1^ in PBS) and incubated at room temperature for 30 min. Fixed, stained cells were analysed by flow cytometry using a Beckman Coulter XL analyzer, with a 488 nm argon ion laser, and results were analysed using System II Software (Beckman Coulter, Hialeah, FL, USA).

### Cell synchronisation

Cells were synchronised by seeding in 100 mm dishes in growth medium, allowing attachment (5–7 h), and replacing the medium with 0.1% (for gastric cancer cells) or 1% FBS (for murine cell lines) in MEM for 72 h. The medium was again replaced with 10% FBS-MEM for 24 h before the addition of 9 *μ*M aphidicolin for 16 h. The cells were washed in PBS and re-fed with complete medium for varying release times before UVC or mitomycin C treatment.

### Rate of DNA synthesis

The S-phase (S) checkpoint was detected by measuring DNA synthesis by a method similar to that described previously (Paulovich and Hartwell, 1995). Briefly, 1 × 10^5^ cells from a growing culture were seeded in 60-mm tissue culture dishes with 3 ml of medium and allowed to grow for more than one doubling. Cells were then pre-labelled in medium containing 10 nCi of [^14^C]thymidine and 0.5 *μ*M cold thymidine. Pre-labelling provides an internal control for cell number by allowing normalisation for the total DNA content of samples. Before irradiation, the medium was replaced with pre-warmed medium and cells were exposed to 3, 5 or 10 J m^−2^ at room temperature and returned to 37°C. [^3^H]thymidine at 0.5 *μ*Ci was added for 30 min at different times after UVC. The cells were then collected and the rate of DNA synthesis for each sample was calculated as ^3^H/^14^C d.p.m. and presented as a percentage of the control values obtained from sham-irradiated cells at the same time point, as described previously (Zhou *et al*, 2002).

The rate of DNA synthesis was also examined by a BrdU incorporation method. Fhit +/+ and −/− cells were incubated at 37°C for 24 h, and then mock-treated or treated with UVC at indicated doses. After 30 min, 10 *μ*M BrdU (Sigma) was added for 1 h. Cells were then harvested and fixed in 70% ethanol at 4°C overnight. Samples were washed twice with PBS, incubated in 2 N HCl/0.5% Triton X-100 (v v^−1^) at room temperature for 30 min, followed by 0.1 M sodium borate for 2 min and washed twice with PBS containing 0.5% Tween 20/0.5%BSA. After that, samples were incubated for 20 min with 2 *μ*l of anti-BrdU-FITC (Pharmingen). After being washed twice with PBS containing 0.5% Tween 20/0.5%BSA, cells were incubated for 30 min with 100 *μ*g ml^−1^, RNase and 10 *μ*g ml^−1^ PI, and analysed by flow cytometry. Cell staining using anti-mouse IgG1 FITC-labelled antibody was used to set the background green fluorescence. At least 10 000 events were gated for each experiment.

### Mutation frequency

Fhit−/− and −/− UV1 were seeded in T175 flasks (1.65 × 10^8^ cells total) in a medium containing 6-thioguanine (6TG, 3 × 10^−^6 M) and cultured for 3 weeks. The number of surviving colonies per 10^7^ cells was calculated for each cell type.

### Western blot analysis

Protein was prepared by lysing cells in a solution of 30 mM Tris (pH 7.5), 10% glycerol, 150 mM NaCl and 1% NP40 with proteinase inhibitors. Approximately 75 *μ*g of total protein was electrophoresed on 12% SDS–PAGE gels, transferred to membrane and blocked in 5% dry milk for 1 h before incubation with primary antibodies against Chk1 (sc-8408, Santa Cruz Biotechnology Inc, Santa Cruz, CA, USA), Phospho-Chk1 (23415, Cell Signaling Technology Inc, Beverly, MA, USA), Cdc25A (sc-7389, Santa Cruz Biotechnology Inc), GAPDH (MAB374, Chemicon International, Temecula, CA, USA) or Fhit (Fong *et al*, 2000). Membranes were incubated in the appropriate secondary antibody labelled with HRP and the signal detected with Super Signal Chemi-luminescent Substrate (Pierce, Rockford, IL, USA).

## RESULTS

### Characteristics of Fhit-positive and -negative cells

**Figure 1:**
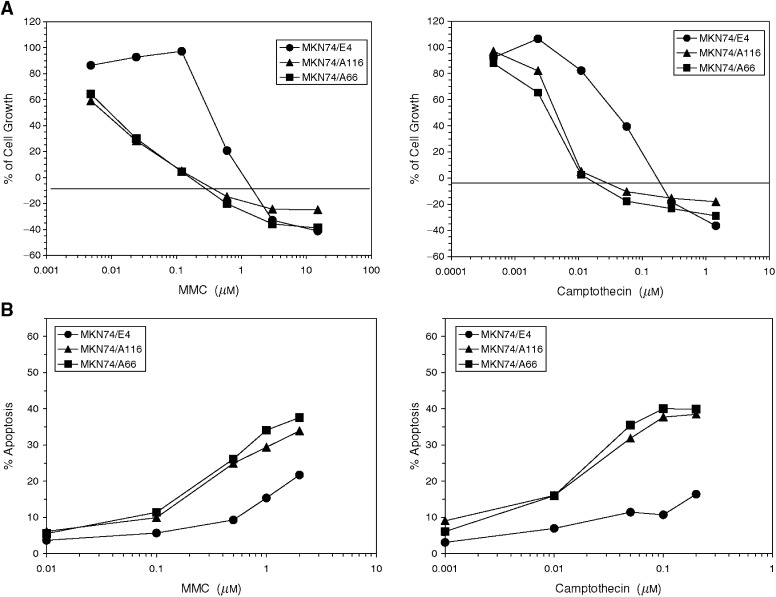
Effect of genotoxic agents on cell growth and apoptosis induction in Fhit-positive (MKN74A66 and A116) and Fhit-negative (MKN74E4) gastric tumour cell lines. (**A**) Cell growth after 72 h exposure to mitomycin C or camptothecin, determined by MTS tetrazolium dye conversion assay. (**B**) Induction of apoptosis after 24 h exposure to mitomycin C or camptothecin determined by TUNEL assay.

Cancer cell clones selected for expression of exogenous Fhit show similar *in vitro* growth characteristics when compared to sister clones transfected with vector only (Siprashvili *et al*, 1997). Likewise, normal kidney cells from Fhit +/+ and −/− mice, established in culture, show similar doubling times and growth factor requirements (data not shown). So what sort of function could Fhit be involved in if its effect is observed through suppression of growth *in vivo* but not *in vitro*? One possibility is that Fhit could be involved in responses to external signals, such as stress inducers, and a number of agents were tested, including staurosporine, peroxide, hypoxia, ultraviolet and ionising radiation, and cytostatic and genotoxic drugs. We first examined MKN74 gastric carcinoma cells, clone MKN74E4, that lack Fhit expression due to homozygous deletion of coding exons, in comparison to MKN74A66 and A116 cells expressing exogenous *FHIT* cDNA. Genotoxic agents, mitomycin C and camptothecin induced dose-dependent decreases in cell growth in all the three cell lines; however, the Fhit-positive cells were significantly more sensitive than the Fhit-negative cells (Figure 1A

). At the IC_50_ concentration, these differences were 36- and eight-fold for mitomycin C and camptothecin, respectively. Consistent with the cell growth data, a greater increase in induction of apoptosis due to these compounds was observed in the Fhit-positive cells than in the Fhit-negative cells, at each concentration tested (Figure 1B, TUNEL assay). The differences tended to be greater at higher drug concentrations, as apoptosis was measured after 24 h drug exposure, rather than after 72 h, as was carried out for the cell survival assays.

**Figure 2:**
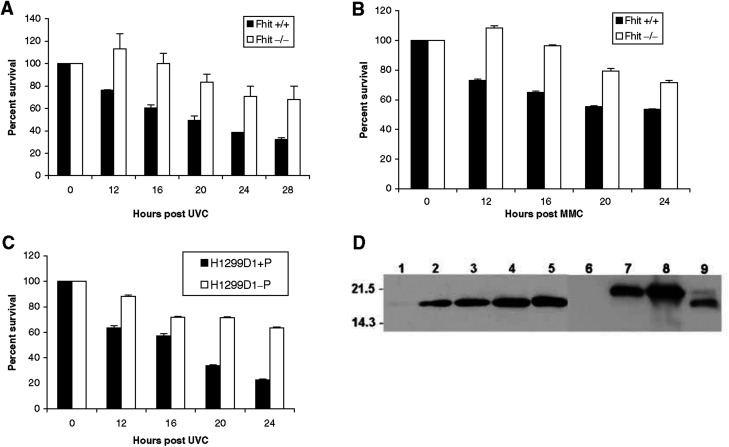
Enhanced survival of Fhit-deficient cells after UVC and mitomycin C treatment. (**A**) Graphic representation of the percent of cells surviving after 60 J m^−2^ UVC treatment. Fhit-positive cells undergo UVC-induced apoptosis, as seen in the decreasing percent of surviving cells. Fhit-negative cells do not show a decrease in survival until 20 h after UVC treatment, and show two-fold more surviving cells by 28 h after treatment (*P*<0.0001, for difference between the decrease in survival of +/+ and −/− cells at 28 h). (**B**) Fhit +/+ and −/− cells were treated with 5 *μ*M mitomycin C and surviving fractions determined. Fhit +/+ cells demonstrate a gradual decrease in survival over time, whereas Fhit −/− cells continue to proliferate before a more gradual decrease in survival. (Two-tailed *t*-test reveals that decrease in survival at 24 h is significant (*P*=0.0079).) Survival experiments were in duplicate or triplicate. Two-tailed Fisher's exact test computed at http://www.matforsk.no/ola/fis her.htm. (**C**) H1299D1+P (Fhit positive) and H1299D1-P (Fhit negative) cells were exposed to 60 J m^−2^ UVC. Fhit-positive cells died rapidly while Fhit-negative cells show a survival rate >60% at 24 h post UVC (*P*<0.0001 for survival difference at 24 h). (**D**) Expression of Fhit protein in Fhit-transfected cells. Lane 1: H1299D1, no induction; lanes 2–5: H1299D1 after Ponasterone A induction for 16, 24, 48, 96 h, respectively; lane 6: MKN74E4; lane 7: MKN74A66; lane 8: MKN74A116; lane 9: human lung tissue. The higher Fhit band in lanes 7 and 8 is a result of the FLAG tag (Siprashvili *et al*, 1997). Lane 9 shows a small amount of phosphorylated Fhit (Pekarsky *et al*, 2004).

**Figure 3:**
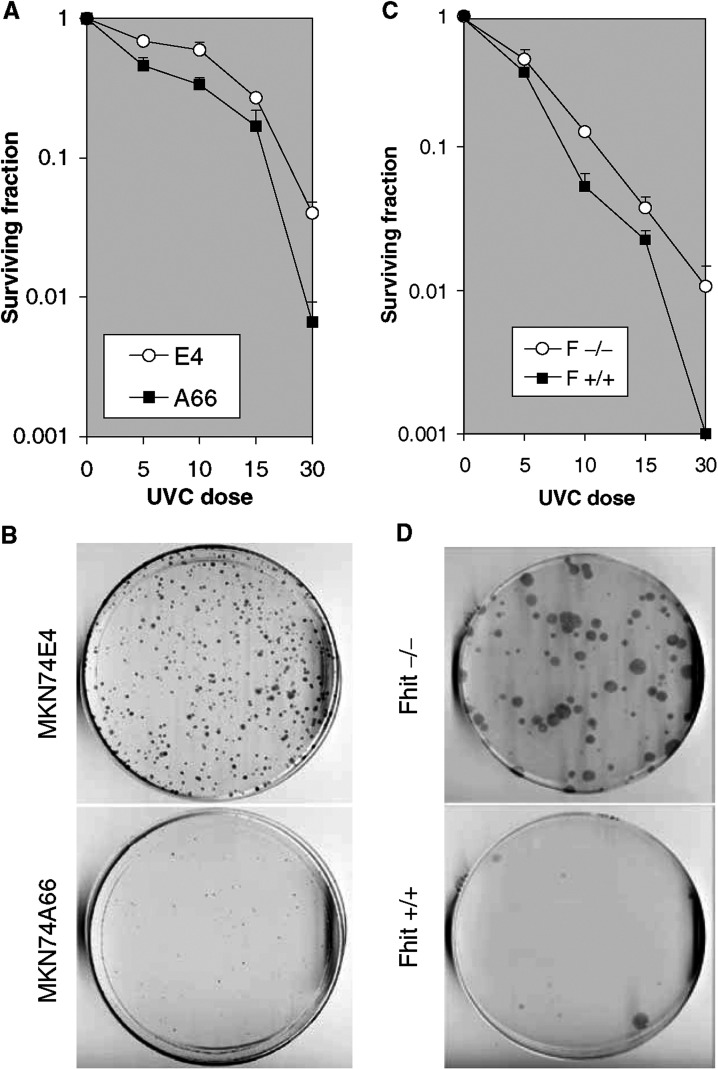
Clonigenicity of UVC-treated Fhit-positive and -negative cells. (**A**) Dose–response clonigenicity for Fhit-positive and -negative A66 and E4 cells, respectively. MKN74E4 and A66 cells were seeded (3000 cells dish^−1^) after no treatment, or 5, 10, 15 and 30 J m^−2^ UVC. After 14 days, cells were fixed in methanol and stained with Giemsa. The values plotted are the averages from two separate experiments, containing three replica plates each. (**B**) MKN74E4 (Fhit negative) and MKN74A66 (Fhit positive) cells were exposed to 60 J m^−2^ UVC, collected, counted and re-seeded (7500 per dish). After 18 days plates were fixed in 1 : 1 methanol : acetone, stained with Giemsa and colonies counted. E4 cells formed an average of 300 colonies/7500 cells and A66 an average of 30 colonies/7500 cells. The absence of Fhit confers an advantage in surviving the lethal dose of UVC. (**C**) Dose–response clonigenicity for Fhit +/+ and −/− mouse cells. The cells were seeded (3000 cells dish^−1^) after no treatment or 5, 10, 15 or 30 J m^−2^ UVC. After 14 days, the cells were fixed and stained. The averages from two separate experiments, containing three replica plates each, are shown. (**D**) Fhit +/+ and −/− cells were exposed to 60 J m^−2^ UVC, collected, counted and re-seeded to dishes (5000 per dish). After 18 days, plates were fixed in 1 : 1 methanol : acetone, stained with Giemsa and colonies counted. Fhit −/− cells formed an average of 90 colonies/5000 cells and +/+ cells an average of 10 colonies/5000 cells.

Enhanced survival of the Fhit −/− normal mouse kidney cells after UVC light treatment was the first evidence for an *in vitro* phenotypic effect of Fhit deficiency in otherwise normal cells (Figure 2A

). The MKN74A66 and E4 cells were then treated with 60 J m^−2^ UVC and the Fhit-negative E4 cells were found to be UVC resistant in a clonigenicity assay (Figure 3B

), while the Fhit −/− cells were also found to be mitomycin C resistant in viability assays (Figure 2B). Human lung carcinoma cells, H1299 (Fhit− and p53−), were transfected with the ecdysone-inducible vectors, pVgRXR and pIND-Fhit. Clone H1299D1 was cultured with (Fhit+) or without (Fhit−) Ponasterone A, treated with 60 J m^−2^ UVC and percent survival calculated after various times. More than twice as many Fhit− cells survived 20 and 24 h post UVC (Figure 2C). Expression of Fhit protein in Fhit-transfected cells used for these studies is shown in Figure 2D.

A clonigenicity assay showed that after high-dose UVC treatment, ∼10-fold more colonies grew on the plates seeded with Fhit-deficient normal mouse cells and the Fhit-deficient gastric cancer cells (Figure 3B, D), although the positive and negative cell pairs have similar plating efficiencies. Dose–response curves for inactivation of colony formation by UVC are shown in Figure 3A, C. The slopes of the lines for relative colony formation *vs* UVC dose are an indication of the effect of Fhit expression on UVC sensitivity in murine kidney and human gastric cancer cells. Colonies were selected from the Fhit −/− and +/+ dishes of UVC surviving clones, and were tested in a second exposure to 60 J m^−2^ UVC to determine if surviving clones had developed mutations that caused UVC resistance. The surviving clones showed UVC sensitivities similar to the parental Fhit +/+ and −/− cell lines (data not shown). Additionally, karyotypes of the UVC survivor clones exhibited near-diploid karyotypes (model chromosome number 42.6), very similar to the near-normal (model chromosome number 42) karyotype of the parental Fhit −/− cells; the majority of Fhit −/− cells before and after UVC exhibited trisomy 19 and some cells showed trisomy 12 and/or marker chromosome(s) (data not shown).

**Figure 4:**
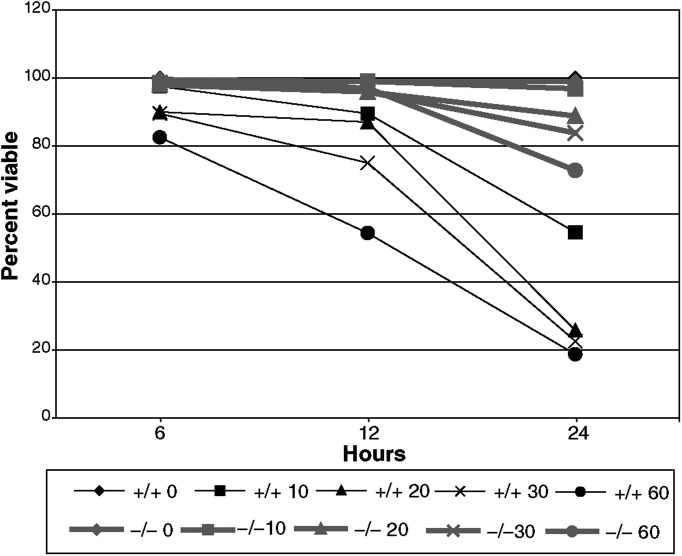
Dose response of Fhit +/+ and −/− cells to UVC treatment. Fhit +/+ and −/− cells were treated with various doses of UVC, as indicated on the graph legend, collected and stained with trypan blue to determine the fraction of viable cells at each time point. At doses of 20 J m^−2^ or higher, Fhit +/+ cells consistently demonstrate lower viability.

A dose–response curve (Figure 4

) for Fhit-deficient murine cells showed that even at 10 J m^−2^ UVC there was a large difference in viability of Fhit −/− and +/+ cells 24 h after treatment.

### Cell cycle analyses

**Figure 5:**
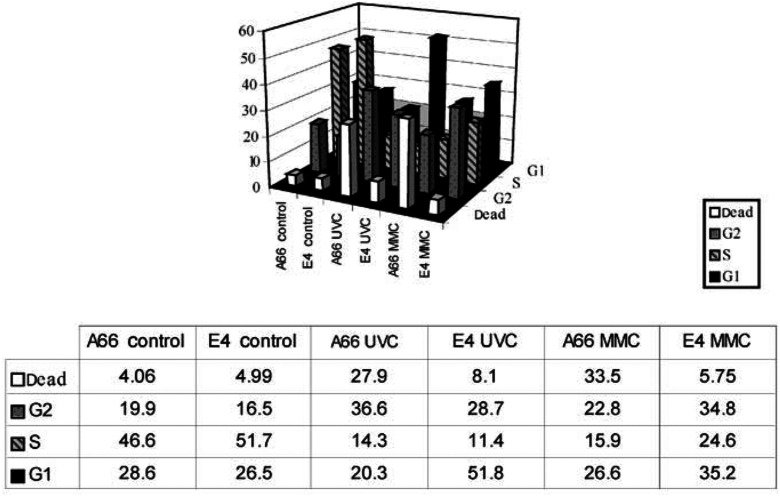
UVC and mitomycin C induced cell death in Fhit-positive but not in Fhit-negative cells. Cells were synchronised first by growth in low serum and then by aphidicolin treatment for 16 h. Cells were released into S phase for 3–5 h and then left untreated (control) or mitomycin C or UVC treated. FACs analysis was performed at 18–21 h afterward, as shown for a representative experiment (21 h). The fraction of cells in specific phases of the cell cycle are illustrated in the bar graph and listed in the table below the bar graph.

To clarify biological events occurring after exposure to UVC or mitomycin C, we compared cell cycle profiles of Fhit-positive cells to the respective Fhit-deficient murine or human cells, with and without treatment. In pilot experiments, Fhit-deficient human and mouse cells consistently showed ∼10% more cells in S phase and ∼10% fewer cells in G1, under normal growth conditions (not shown); after UVC or mitomycin C, the main difference observed in the cell cycle profiles was that up to 40% of the Fhit-positive cells were sub-G1 in DNA content (apoptotic/necrotic) relative to fewer than 10% of the Fhit-deficient cells, both mouse and human, by 20 h after UVC treatment of mouse or mitomycin C treatment of human or vice versa (not shown). This implied that Fhit-deficient cells remained in S and G2/M phases of the cell cycle longer after stress exposure, while Fhit-positive cells cycled more rapidly and damaged cells became apoptotic. To investigate the cell cycle profiles following stressful exposures, the murine and human cell pairs were synchronised in G1, released into S phase and then treated with UVC or mitomycin C. Results of representative experiments are shown in Figure 5

. After aphidicolin synchronisation and release into S phase, the Fhit-positive and -negative cells exhibit similar profiles with around 50% of cells in S phase at the time of mitomycin C or UVC treatment. At 21 h after UVC (30 J m^−2^), 28% of A66 cells were dead relative to only 8% of E4 cells. Similarly, at 21 h after 5 *μ*M mitomycin C exposure, 33.5% of A66 cells were dead compared to only 6% of E4 cells (see Figure 5 for details). It is also notable that E4 cells show an increase in the G1 fraction to 52% at 21 h post UVC, suggesting that cells have successfully passed the G_2_/M block and reentered G1, while in the 21 h UVC-treated A66, the cells that have exited S/G_2_/M went into apoptosis. After 21 h of mitomycin C, on the other hand, the majority of E4 cells seem to be cycling normally.

### DNA synthesis in Fhit-deficient cells

**Figure 6:**
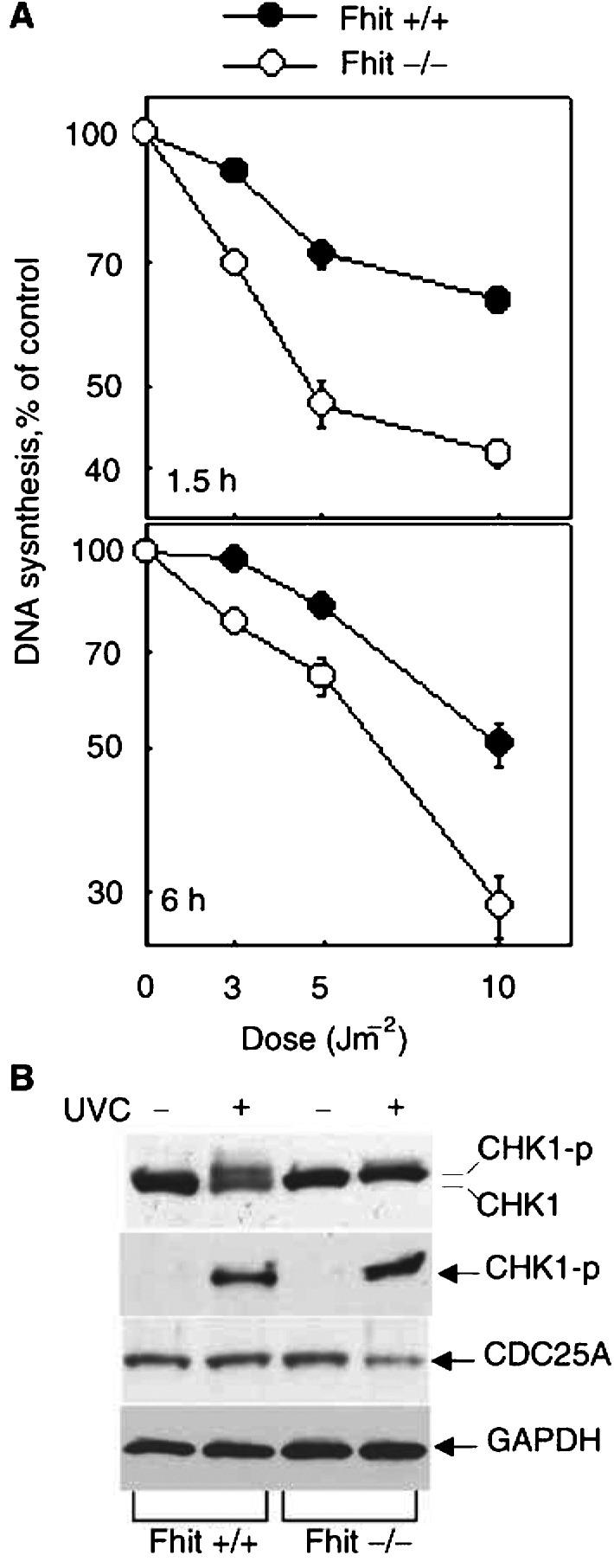
(**A**) The rate of DNA synthesis after UVC treatment in Fhit +/+ and −/− cells. DNA synthesis was examined at 1.5 and 6 h after various doses of UVC treatment in Fhit +/+ and −/− cells. The cells pre-labelled with ^14^C[TdR] were changed with pre-warmed medium before UVC. At 1 and 5.5 h after exposure to different doses of UV, 0.5 *μ*M ^3^H[TdR] was added to the cell culture. After 30 min, the cells were collected and loaded on GF-A filtres set in a Millipore Vacuum chamber. The rate of DNA synthesis for each sample was calculated as ^3^H/^14^C d.p.m. and is presented as a percentage of the control values obtained from untreated cells at the same time point. The data are presented as mean values and standard deviations from three independent experiments. (**B**) The CHK1 pathway is activated in UVC-treated Fhit-positive and -negative cells. The Fhit +/+ and −/− cells were treated with UV (15 J m^−2^) or untreated and harvested 2 h post treatment. Equal amounts of whole-cell extract were immunoblotted with antibodies against Chk1, phospho-Chk1 and Cdc25A, respectively. GAPDH antibody was used as the internal control for equal loading. Similar results were obtained from two independent experiments.

Both UVC and mitomycin C damage engage the *ATR*–*CHK1* DNA damage checkpoint (Paulovich and Hartwell, 1995; Wright *et al*, 1998; Guo *et al*, 2000; Liu *et al*, 2000; Papouli *et al*, 2000; Zhao and Piwnica-Worms, 2001; Lambert *et al*, 2003). Thus, we next investigated the state of DNA synthesis in treated cells. The rates of DNA synthesis in Fhit +/+ and −/− cells early after low doses of UVC irradiation were calculated after ^3^H[TdR] labelling as described (Zhou *et al*, 2002). The rate of DNA synthesis after 3, 5 and 10 J m^−2^ was determined by ^3^H[TdR] labelling assay and results are shown in Figure 6A

. At 6 h after irradiation with 10 J m^−2^ UVC, DNA synthesis in the −/− cells had declined by >70%, while in +/+ cells the reduction was only 45%. From these results, it is clear that the rate of DNA synthesis declines more rapidly and steeply in Fhit −/− cells than in +/+ cells in response to increasing UVC doses. The rate of DNA synthesis determined by BrdU showed a similar tendency (data not shown). At 15 J m^−2^, at 1.5 h after UVC, DNA synthesis was reduced by 60% in −/− cells but by only ∼24% in +/+ cells; at 30 J m^−2^, DNA synthesis was reduced by more than 80% in −/− cells compared to about 50% in +/+ cells. The enhanced inhibition of DNA synthesis in −/− cells occurred at each dose and time tested, whether assessed by the isotopic or BrdU method.

To examine the DNA damage checkpoint in more detail, we next examined expression of Chk1 and Cdc25A, the effectors downstream of Atr in this pathway. As shown in Figure 6B, phosphorylated Chk1 is expressed in both +/+ and −/− cells after UVC treatment and Cdc25A decreased in Fhit −/− cells after UVC, suggesting that the Chkl pathway is activated by UVC in both cell types, with a suggestion of a higher level of phosphorylation of Chk1 in Fhit −/− cells (see Figure 6B), consistent with the dramatic decrease in DNA synthesis.

Experiments to determine the rate of DNA synthesis of the MKN74A66 and E4 cells by BrdU incorporation after UVC treatment showed a decline in DNA synthesis rate of only 20% in A66 cells and 10% in the E4 cells after 60 J m^−2^ UVC (data not shown), suggesting that the DNA replication checkpoint pathways are not intact in these cancer cell clones, although the Fhit-negative E4 cells, like the Fhit −/− murine cells, traversed the S/G_2_/M phases without undergoing apoptosis, while 30–40% of the A66 Fhit-positive cells were apoptotic at 18–20 h after 30 or 60 J m^−2^ UVC, as described above and shown in Figure 5.

### Mutation frequency in UVC survivors

Since Fhit-deficient cell lines are resistant to apoptosis after UVC and mitomycin C treatment, the surviving cells are likely to carry misrepaired DNA damage. We tested this conclusion by growing 1.65 × 10^8^ Fhit-deficient and UVC-surviving Fhit-deficient cells in 6-thioguanine and counting surviving colonies, as an assay for frequency of HPRT mutations. The Fhit −/− cells exhibited an average of 2.7 colonies per 10^7^ cells and UV-surviving UV1 cells exhibited an average of 15 colonies per 10^7^ cells, a difference of 5.6-fold. Similarly, the Fhit-deficient MKN74E4 UV survivor cells showed 2.3-fold more 6TG-resistant colonies than the E4 cells. The differences in mutation frequency at the HPRT locus are consistent with the interpretation that the Fhit-negative UVC-surviving cells have sustained unrepaired or misrepaired DNA damage, which could underlie the susceptibility of Fhit-deficient cells to neoplastic transformation.

## DISCUSSION

A recent review (Skorski, 2002) on oncogenic tyrosine kinases summarised as follows: ‘mutation or overexpression of oncogenes, such as constitutively activated tyrosine kinases, can induce uncontrolled growth, protection from apoptosis, enhanced repair of DNA lesions, prolonged activation of cell-cycle checkpoints providing more time for repair of lethal lesions, upregulation of anti-apoptotic members of the *BCL2* family, protecting cells from apoptotic signals. The unrepaired and aberrantly repaired DNA lesions that result from DNA damage can thus accumulate in the oncogene expressing tumour cells, leading to genomic instability and malignant progression.’ The results of the experiments described here suggest the conclusion that loss of Fhit protein function results in acquisition of features similar to those of an oncogene-transformed cell: protection from apoptosis, rapid activation of DNA damage checkpoint and accumulation of mutations due to lesion misrepair. Thus, we considered the idea that Fhit may be involved in signal pathways that intersect with an oncogenic tyrosine kinase signal pathway, similar to the activated Abl tyrosine kinase pathway. We have preliminarily investigated this possibility by determining if inhibition of Abl activity in our Fhit plus and minus cells might abrogate differences in UVC or mitomycin C responses. Inactivation of the Abl kinase by STI571 (Gleevec) treatment of Fhit-positive and -negative cells did not alter the response to mitomycin C or UVC, suggesting that Abl tyrosine kinase is not part of the Fhit pathway (unpublished data).

As also pointed out by Skorski, oncogenic tyrosine kinases make good targets for antitumour treatments and some such treatments are in clinical trials. Well-known tumour suppressor genes such as *TP53* and *CDKN2A*, encoding p53 and p16, similarly affect cell cycle checkpoint, apoptotic and repair pathways; absence of the wild-type p53 or p16 proteins has effects on cell cycle kinetics, response to DNA damage and apoptosis, but these proteins are not necessarily good targets for treatment, except through gene therapy, necessitating identification of effectors of tumour suppressor signalling pathways, to define new therapeutic targets.

In an approach to define the Fhit tumour suppressor signal pathway, we have developed *in vitro* models to study the effects of absence of Fhit on cellular stress responses. Of the stress inducers tested (staurosporine, hypoxia, chemotherapeutic agents, detachment from substrate, H_2_O_2_, ionomycin (M Ottey *et al*, unpublished)), UVC and mitomycin C induced significantly decreased levels of apoptosis and increased cell survival in Fhit-negative cells. Both UVC and mitomycin C treatment cause DNA crosslinks, a severe form of DNA damage, and both agents induce the DNA damage checkpoint initiated by the activation of Atr and Chk1 kinases (Guo *et al*, 2000; Liu *et al*, 2000; Papouli *et al*, 2000; Zhao and Piwnica-Worms, 2001; Heffernan *et al*, 2002; Unsal-Kacmaz *et al*, 2002). In addition, the topoisomerase I poison, camptothecin, which produces single-strand DNA breaks, also results in the activation of the Atr/Chk1-regulated S-phase checkpoint (Wang *et al*, 2002). This DNA-damaging agent likewise induced apoptosis in MKN74A66 Fhit-positive cells, but less so in E4 Fhit-negative cells.

*ATM* and *ATR* genes are both activated by DNA damage, with *ATM* responding mainly to double-strand breaks, such as induced by ionising radiation, and *ATR* reacting to UV or stalled replication forks (for a review, see Yang *et al*, 2003). Specifically, *ATR* functions in UVC-induced signalling and apoptosis (Wright *et al*, 1998; Heffernan *et al*, 2002). The ATR pathway appears intact in Fhit-deficient cells and there is some suggestion that the *ATR* pathway is, in fact, enhanced in Fhit-deficient mice. For example, DNA synthesis is more rapidly inhibited in Fhit −/− cells after low UVC doses.

The unique phenotype of Fhit-deficient cells, with their rapid and prolonged block in S/G2 after UVC or mitomycin C treatment, coupled with drastically reduced apoptotic response, suggests that Fhit loss also contributes to multiple cell programs that are important in the development of precancer and cancer. The result of the alterations to the DNA damage response is that Fhit-deficient cells escape their legitimate death sentence, with some of them carrying unrepaired or misrepaired DNA lesions that will contribute to further clonal expansion, as shown by the transformation and increased HPRT mutations exhibited by the UVC-surviving colonies.

In fact, loss of most genes involved in DNA damage checkpoints or DNA damage repair, such as *ATM*, *ATR*, *BRCA1*, *BRCA2*, *Fanconi anemia* genes, lead to increased sensitivity to genotoxic agents. All of these can be considered as tumour suppressor genes whose loss leads to increased DNA damage. Loss of Fhit leads to reduced sensitivity to the DNA crosslinking agents UVC and mitomycin C. If Fhit were a checkpoint gene, then we would not expect to see the rapid inhibition of DNA synthesis in Fhit −/− cells after UVC treatment (Figure 6A). Perhaps Fhit protein actually slows the response to UVC by, for example, binding to Hus1 or another member of the 9-1-1 complex (Roos-Mattjus *et al*, 2002; Weiss *et al*, 2002) to slow movement into the nucleus. This may prevent a too drastic response to low-dose genotoxic agents. But Fhit protein must have an effect also at the G_2_/M checkpoint when assessment of DNA damage repair takes place before re-entering the cell cycle. At this point, after 30–60 J m^−2^ UVC, the Fhit-positive cells are correctly assessed as harbouring unrepaired DNA that triggers apoptosis, while the similarly treated Fhit-negative cells go through mitosis and back into G1, still carrying improperly repaired or unrepaired DNA lesions, as suggested by the higher mutation rate of the Fhit −/− UV survivors. There are no reports of other genes for which inactivation enhances cell survival after UVC exposure. We are currently examining in detail the proteins involved in progression through late S/G_2_/M, to determine where signal pathways may diverge in Fhit-positive and -negative cells.

We have known for some time that Fhit protein is, directly or indirectly, proapoptotic (Ji *et al*, 1999; Sard *et al*, 1999; Dumon *et al*, 2001; Ishii *et al*, 2001), so perhaps the difference in Fhit-positive and -negative cells at the G_2_/M boundary is not in checkpoint or cycling defects but in sending or responding to an apoptotic signal. Very recently, it was reported that Noxa-deficient mice show decreased DNA damage-induced apoptosis in fibroblasts (Shibue *et al*, 2003; Villunger *et al*, 2003) and Noxa, a BH3-only member of the BCL2 family, is considered a critical mediator of the apoptotic response induced by p53 (Oda *et al*, 2000). Noxa was not required for normal development since nullizygous mice were born at the expected frequency and the mice appeared normal. The role of Noxa in stress-induced apoptosis was investigated in primary embryo fibroblast cultures by treating the MEFs with etoposide (CPT11), *γ*-radiation and other agents. Noxa-deficient MEFs exhibited modest resistance to etoposide-induced apoptosis, although cancer susceptibility has not yet been assessed. Also, Noxa −/− mice showed resistance to X-ray-induced gastrointestinal death, with reduced apoptosis of epithelial cells of small intestine crypts (Shibue *et al*, 2003). Thus, Noxa-deficient mice may have a phenotype similar to Fhit-deficient mice (Hu *et al*, 2004), suggesting that the Fhit proapoptotic function may involve the Noxa pathway.
